# Use of a Septal Stapler to Secure a Septal Free Mucosal Graft to the Nasoseptal Flap Donor Site Following Endoscopic Endonasal Resection of a Pituitary Adenoma [Fn FN24apr0016-1]

**DOI:** 10.1055/a-2531-6140

**Published:** 2025-04-23

**Authors:** Jessa E. Miller, Jakob L. Fischer, Marilene B. Wang

**Affiliations:** 1 Department of Head & Neck Surgery, University of California, Los Angeles, California, United States

**Keywords:** nasoseptal flap, skull base reconstruction, free mucosal graft, pituitary adenoma

## Abstract

There are multiple techniques that can be utilized to reconstruct skull base defects following endoscopic endonasal pituitary surgery. The nasoseptal flap (NSF) is a vascularized reconstructive option that is commonly used to repair skull base defects. There are several factors that must be considered when deciding to perform a NSF. If a NSF is harvested from one side, the posterior septal mucosa on the contralateral side is often sacrificed and wasted during the posterior septectomy for the endoscopic endonasal pituitary approach. Harvesting a NSF from one side and a posterior septal free mucosal graft (FMG) from the contralateral side affords the surgeon multiple reconstructive options depending on the size of the defect and the presence of a CSF leak at the end of the tumor resection. After a NSF is performed, patients often have significant crusting along the exposed anterior cartilaginous septum that results from delayed remucosalization. If a contralateral posterior septal FMG is harvested, this can be secured to the caudal septal NSF donor site to help minimize postoperative sinonasal symptoms, principally caudal septal crusting which can result in nasal obstruction. In this operative video, we demonstrate the use of a septal stapler to secure a FMG to the anterior NSF donor site following endoscopic endonasal resection of a pituitary adenoma. The staples are dissolvable, and the septal stapler technique is more efficient than performing a quilting suture.

**Video 1** This video demonstrates the use of a septal stapler to secure a septal FMG to the NSF donor site following endoscopic endonasal resection of a pituitary adenoma.


